# HIV/STI Prevention Strategies During COVID-19 Among PrEP-Eligible Cisgender Women in New York State: A Qualitative Analysis

**DOI:** 10.3390/ijerph23040500

**Published:** 2026-04-14

**Authors:** James M. McMahon, Natalie M. Leblanc, Janie E. Simmons, Keosha Bond, Whitney Irie, Danielle C. Alcena-Stiner, Lindsay Batek, Chen Zhang

**Affiliations:** 1 School of Nursing, University of Rochester Medical Center, Rochester, NY 14642, USA; natalie_leblanc@urmc.rochester.edu (N.M.L.); danielle_alcena@urmc.rochester.edu (D.C.A.-S.); lindsay_batek@urmc.rochester.edu (L.B.); chen_zhang@urmc.rochester.edu (C.Z.); 2 National Development and Research Institutes-USA, New York, NY 10001, USA; jsimmons@ndri-usa.org; 3 Department of Community Health and Social Medicine, School of Medicine, City University of New York, New York, NY 10031, USA; kbond@med.cuny.edu; 4 School of Social Work, Boston College, Chestnut Hill, MA 02467, USA

**Keywords:** HIV/STI prevention, women’s sexual health, relationship dynamics, COVID-19 pandemic

## Abstract

**Highlights:**

**Public health relevance—How does this work relate to a public health issue?**

- PrEP-eligible cisgender women continue to face HIV/STI vulnerability while underutilizing PrEP, making it important to understand how prevention is actually practiced within real-world contexts.
- The COVID-19 pandemic disrupted partnering opportunities and access to sexual health services, reshaping prevention decision-making and highlighting gaps in testing and PrEP access for women.

**Public health significance—Why is this work of significance to public health?**

- This study describes dynamic “prevention ecologies,” showing how women combine and adapt condoms, HIV/STI testing, PrEP use or discontinuation, partner negotiation, and at times abstinence as relationship contexts, perceived risk, and access constraints change over time.
- The study identifies meaningful differences in prevention strategies by race/ethnicity, age, and site, including episodic PrEP use during uncertainty or condom-negotiation challenges.

**Public health implications—What are the key implications or messages for practitioners, policy makers and/or researchers in public health?**

- Prevention counseling and programs should be women-centered and culturally/contextually tailored, explicitly supporting flexible, relationship-responsive combination prevention rather than single-method messages.
- Status-neutral approaches that routinize HIV/STI testing (including self-testing) and ensure low-barrier access to PrEP/PEP—while addressing partner resistance, stigma, and geographic inequities—are needed to align services with women’s lived prevention strategies.

**Abstract:**

PrEP-eligible cisgender women underutilize PrEP, and little is known about how PrEP fits within broader HIV/STI prevention ecologies shaped by personal preferences and relationship contexts. These ecologies inform prevention strategies that shift with relationship dynamics and vary by race/ethnicity, age, and setting; the COVID-19 pandemic further influenced these contexts. Data were drawn from the Women’s Study in Sexual Health and Empowerment (WISE), a mixed-methods study of PrEP-eligible cisgender women in New York City and Rochester, NY. One-time semi-structured interviews were conducted with 48 women from the WISE cohort. Women described tailoring prevention to relationship context, often initiating relationships with condoms and later relying more on trust and periodic HIV/STI testing. Strategies included situational condom use, combination prevention with PrEP and HIV/STI testing, and PrEP initiation or discontinuance as perceived risk changed. Women also reported challenges negotiating condom use, including partner resistance, and some described abstinence as a deliberate strategy. Comparative analyses identified patterns by race/ethnicity, age, and site. Pandemic-related disruptions reduced opportunities for new partnerships, altered relationship dynamics, and shifted some prevention conversations toward SARS-CoV-2 exposure risk. Findings highlight the need for women-centered, culturally and contextually tailored prevention services that strengthen PrEP access and routinized HIV/STI testing while accounting for relationship dynamics.

## 1. Introduction

Despite declines in HIV incidence over the past decade, cisgender women in the United States remain substantially affected by the epidemic. Women account for roughly one in five new HIV diagnoses nationally, and most infections among women are attributed to heterosexual contact, with a smaller proportion related to injection drug use [1]. Black and Latina women bear a disproportionate burden: they comprise the majority of women living with HIV and of new diagnoses among women, even though they represent a minority of the U.S. female population [1,2]. These disparities reflect longstanding social and structural inequities, including racism, poverty, housing instability, and unequal access to high-quality health care and prevention services [3,4].

Women’s HIV and STI prevention options include condom negotiation, partner discernment and assessment, regular HIV/STI testing, post-exposure prophylaxis (PEP), and oral and long-acting pre-exposure prophylaxis (PrEP). Prior studies have shown that women often rely on more than a single prevention strategy; instead, they combine methods and adapt them over time, negotiating condoms more consistently with casual partners than with primary partners, seeking testing at key relationship transitions, or temporarily abstaining from sex during periods of heightened uncertainty or risk [5,6,7,8,9]. Yet these strategies are constrained by gendered power dynamics, economic dependence, and caregiving responsibilities, which can limit women’s ability to insist on condoms, disengage from unsafe relationships, or prioritize their own sexual health [10,11].

The expansion of biomedical prevention, particularly PrEP, has created new opportunities but also new gaps. Nationally, only a small fraction of PrEP users are women, despite women accounting for nearly one-fifth of new HIV diagnoses [1,12,13,14]. Recent reviews and implementation studies consistently document low awareness of PrEP among cisgender women, limited perceived personal HIV risk, stigma around HIV and PrEP use, medical mistrust and experiences of medical distrust—especially among Black and Latina women [15,16,17]. Policy and system-level barriers, such as lack of Medicaid expansion, prior authorization requirements, fragmented sexual and reproductive health services, and limited provider knowledge or comfort discussing PrEP with women, further reduce access [17,18,19]. At the same time, many women continue to rely primarily on condom negotiation and partner comfort with condoms, monogamy expectations, and periodic HIV/STI screening, indicating that PrEP is being layered onto, rather than replacing, existing prevention practices [20,21,22].

Racial and ethnic inequities are deeply intertwined with these prevention patterns. Studies focusing on Black and Latina cisgender women underscore how structural racism, neighborhood disadvantage, and under-resourced health systems shape both exposure to HIV risk and the availability of prevention resources [11,17,23]. Women in these communities often encounter stigmatizing or dismissive treatment in health care settings, experience unmet mental health and social support needs, and face logistical barriers such as transportation, childcare, and inflexible work schedules, constraining access not only to PrEP but also to routine HIV/STI testing, contraception, and broader sexual health services [3,24].

Relationship and gender-power dynamics further shape how women navigate HIV/STI prevention: across studies, condom use is more common early in relationships and with non-primary partners and often declines as trust, emotional intimacy, or expectations of monogamy increase; women frequently describe difficulty negotiating condoms with male partners who resist their use or interpret them as a sign of mistrust, and intimate partner violence can directly undermine women’s ability to insist on condoms, attend clinics, obtain testing, or initiate PrEP [7,10,25,26,27]. The COVID-19 pandemic added another layer of complexity, as lockdowns and service disruptions limited access to in-person HIV/STI testing, contraceptive care, and PrEP clinics, while telehealth and home HIV/STI self-testing emerged as alternative modes of care; emerging evidence suggests that these disruptions widened existing inequities in prevention access, particularly for women of color and women with caregiving responsibilities [28,29,30].

Against this backdrop, much of the recent literature on cisgender women and HIV prevention is anchored in PrEP—examining awareness, willingness, and uptake; testing clinic-based, community-based, and digital interventions; and mapping policy and implementation barriers [18,20,31]. Fewer studies have taken a holistic view of women’s HIV/STI prevention “ecologies,” encompassing how they combine and sequence multiple strategies, including condoms, partner assessment, HIV/STI testing, PrEP/PEP, and abstinence [32]. The Women’s Study in Sexual Health and Empowerment (WISE) was designed to address these gaps by examining how women describe their HIV/STI prevention strategies with different types of male partners, how they balance combination prevention methods, and how race and ethnicity, age, relationship dynamics, and pandemic-related contexts shape these decisions.

## 2. Materials and Methods

### 2.1. Study Overview

The Women’s Study in Sexual Health and Empowerment (WISE) is a mixed-methods longitudinal cohort study designed to explore the multilevel factors contributing to racial/ethnic disparities in healthcare utilization and sexual health outcomes, with a primary focus on HIV and STI prevention. The WISE study was guided by the Gelberg-Andersen Behavioral Model for Vulnerable Populations [33] and the Gender and Assets approach [34], which were selected because together they address both structural and interpersonal determinants of health, including access to care, social vulnerability, gendered relationship power, and resource-based influences on prevention decision-making. In the present qualitative analysis, these frameworks were used primarily to guide interview guide development, purposive sampling, and interpretation of findings rather than as discrete analytic models applied separately. Intersectionality informed the design of the interview guide, purposive sampling strategy, and comparative analyses by directing attention to the ways HIV/STI prevention decision-making may vary across intersecting social positions and power relations, particularly race/ethnicity, relationship context, and gendered relationship power [34,35,36,37]. Empowerment informed the study by focusing inquiry on women’s perceived agency, negotiation processes, and access to social, economic, and relational resources relevant to prevention decision-making [34]. Guided by these frameworks, the interview guide was designed to illicit women’s narratives about predisposing, enabling, and need-related determinants of HIV/STI prevention (including structural and social vulnerability) and gendered power dynamics and asset-based resources (e.g., social, economic, and relational assets) that shape prevention decision-making. Data were collected between January 2020 and August 2021 from adult cisgender women living in New York City and Rochester, NY.

### 2.2. Study Participants

The study sample included self-reported HIV-negative adult women who reported behaviors that increased their exposure to HIV or STIs. To participate, individuals had to meet the following eligibility criteria: (a) assigned female at birth and identifying as a woman; (b) age 18 or older; (c) fluent in English or Spanish; (d) self-reported HIV-negative status; (e) resident of the NYC or Rochester metro area; (f) willing and able to provide informed consent; and (g) currently using PrEP or exhibiting behaviors consistent with PrEP indications, including having a sexual partner who was HIV-positive, of unknown HIV status, or anonymous; exchanged sex for money or drugs; used illicit substances during sex in the past 3 months; injected drugs in the past 6 months; or was prescribed post-exposure prophylaxis (PEP) more than once in the past year.

### 2.3. Participant Recruitment

For the qualitative component, participants from the larger WISE cohort were selected using purposive sampling to ensure diversity across racial and ethnic identities, healthcare settings, sexual behaviors, and PrEP use. Individuals selected through this sampling approach were contacted by email to solicit interest in participating in a qualitative interview. During recruitment and consent, participants were informed that the interviews were intended to explore women’s HIV/STI prevention strategies with sexual partners and that the knowledge gained would help inform culturally and contextually tailored prevention services for women.

### 2.4. Quantitative Data Collection

The WISE health survey was administered using the QDS-Web platform (Questionnaire Development System software, ver. 4.0.0.4), available on both mobile devices and computers. Surveys were available in English or Spanish and encompassed a variety of domains, including demographics, mental and physical health, sexual behavior, substance use, HIV and STI history, healthcare access, discrimination, and sexual empowerment. All survey data were securely stored on a HIPAA-compliant server and later transferred to a secure university server for analysis using SAS software (vers. 9.4).

### 2.5. Qualitative Data Collection

Forty-eight one-time semi-structured interviews were conducted via Zoom (mean duration: 43 min), guided by an interview protocol covering topics such as sexual health and health care access and utilization, sexual behavior and relationship history, use of HIV/STI prevention methods, including PrEP/PEP, and COVID-19 pandemic experiences. Interviews were conducted by JES (NYC) and NML (Rochester), two female members of the research team who were experienced qualitative interviewers and played principal roles in developing the interview guide. The interviewers had no prior relationship with participants. All interviews were conducted in English, based on participant preference, and recorded, transcribed, and imported into Dedoose software (ver. 10.0.34) for analysis. Interviewers also prepared field notes following each interview to document contextual observations and initial analytic impressions. Data collection continued until thematic saturation was reached. Transcripts were not returned to participants for comment or correction.

### 2.6. Qualitative Approach and Thematic Analysis

A narrative approach was employed to understand how participants constructed and made meaning of their personal stories and experiences of HIV/STI prevention over time and within broader social and cultural contexts. The Framework Method was utilized for thematic analysis, providing a structured and systematic way to identify patterns while maintaining the coherence of individual narratives [38]. The analysis began with an in-depth review of the transcripts and field notes, followed by the development of a coding scheme based on the interview guide and six initial transcripts. Fourteen additional transcripts were double-coded to further refine the coding framework, resulting in a final set of 61 hierarchically structured codes. The remaining transcripts were coded by two main coders (JES and JMM), and all transcripts were reviewed to ensure consistency in coding. Coded excerpts were cross-referenced with participants’ survey responses (e.g., demographics, site, PrEP-related measures) and summarized in a case-by-variable matrix to examine how themes clustered and differed across quantitative characteristics.

This structured process facilitated the synthesis of key themes, providing insights into participants’ lived experiences. After coding was completed, we conducted a thorough thematic analysis to identify key themes and sub-themes across the entire dataset. This initial step involved organizing coded excerpts into matrices to assess patterns across major themes related to HIV/STI prevention strategies. Through an iterative process, key themes were refined, and sub-themes were generated to capture nuanced variations. Field notes were used during analysis to provide additional contextual detail and to inform interpretation. We then conducted subgroup comparisons by race/ethnicity (Latina, Black/African American, or White/Caucasian), site (NYC or Rochester), and age group (35 and under, 36–54, and 55 and over) using subgroup-by-theme matrices. We compared how themes were expressed across groups and used descriptive summaries of excerpt patterning as an analytic aid. Interpretations were based on the consistency, depth, and contextual variation of accounts within groups, with attention to overlapping and contradictory cases, rather than on frequency alone. This approach ensured that both shared and subgroup-specific experiences were adequately represented in the analysis. These comparisons were not intended as a formal intersectional analysis of combined identity positions.

## 3. Results

Table 1 describes the characteristics of the sample used in the qualitative analysis.

### 3.1. Thematic Analysis: Overview of Major Themes and Sub-Themes of Women’s HIV/STI Prevention Strategies (Values in the Parentheses After Each Excerpt Indicate Participant’s Age and Race/Ethnicity)

#### 3.1.1. Partner Assessment and Contextual Strategies

A primary theme that emerged involved how women evaluate their partners’ behaviors, health status, and trustworthiness to inform their decisions around HIV/STI prevention. Women often adapt their prevention strategies depending on their relationship with each partner and how they perceive their partner’s health and behaviors. This strategy is particularly salient in distinguishing between casual and primary partners.

1. Use of PrEP or condoms based on partner type

Some women reported using PrEP or condoms only in specific relationships where they felt less certain about their partner’s sexual health, such as with casual or less familiar partners, while feeling more comfortable not using condoms with trusted, primary partners.

*Um, with my primary [partner]—we don’t use any protection [condoms]. Um, but with my other partners, I do [use condoms]. Um, I think with my primary partner, because, um, we just—I don’t know. We just decided that, you know, with each other, we’re not going to use condoms, and then but with the expectation is that with anyone else, we will.*

*(36, AA-African American)*

2. Adjusting prevention based on partner’s behaviors, character, or history

Some women made prevention decisions based on their partner’s health habits (e.g., regular doctor visits or other health-conscious behaviors), trusting that these behaviors reduced the likelihood of HIV or STI transmission. Women also adjusted their prevention strategies based on what they knew about their partner’s sexual behavior or history. For example, if they believed their partner had fewer sexual partners or engaged in behaviors that were perceived as safer, they were more likely to forgo condoms. Women sometimes decided to use condoms in specific situations, such as when they felt uncertain about their partner’s recent activities or when there was doubt about their partner’s fidelity, despite being in a long-term relationship.

*Um, now, the other one [sexual partner], I don’t [use condoms], but that’s because, you know, he’s fresh. You know what I mean? You know, so—and I know that he’s not doing anything… and you know that he’s not doin’ those things. So, um- so that’s how I identify that situation.*

*(36, W-White)*

*Definitely I wanna start using condoms especially, like, with my ex ‘cause I did see him… and we do, like, I guess then have sex. But because I don’t wanna be with him and I know that he’s not the best of guys when it comes to, like, keeping his stuff in his pants I do wanna implement, like, I wanna start using condoms with him, or, like, some sort of like PrEP ‘cause to be honest I don’t trust him.*

*(33, L-Latina)*

3. Perceived safe alternative prevention methods

In some cases, women believed that some sexual practices (e.g., pull-out method) or avoiding sexual practices (e.g., anal sex) or drug use with partners provided adequate prevention against HIV/STI transmission, even if they didn’t use conventional forms of protection.

*But I think I took precaution enough that yeah—you could let it in five minutes that you have sex. But like I didn’t go and do drugs. I didn’t let—do like any anal thing that woulda got me more in trouble or stuff like that. Like, I try to get away from it.*

*(56, L)*

#### 3.1.2. Evolving Relationships: From Protection to Trust

This theme captures the common strategy of beginning new relationships by using condoms and gradually transitioning to relying on trust as relationships become more intimate and committed. As trust grows, women feel comfortable discontinuing condom use, relying instead on mutual monogamy or regular HIV testing as their protection method.

1. Initial condom use followed by discontinuation

Many women reported consistently using condoms at the beginning of their relationships, especially when the relationship was new or casual. As the relationship progressed and became more serious or committed, they gradually discontinued condom use, relying on trust and mutual monogamy for protection.

*Yeah, so we usually—um, when we first started dating, he-he like wore by default, used condoms, which was, um, what he did, and then after like—being committed, and then, um, kinda, like I did testing and stuff, we decided to like not use condom just because we’re-we’re primary partners, and we’re not having sexual relationship with other people. Um, that’s been working quite well right now.*

*(20, W)*

2. Trust as a primary factor for discontinuing condom use

Condom discontinuation was often shaped by a growing sense of trust in the relationship. As relationships became more established, some women described relying on perceived honesty, exclusivity, and personal comfort to judge whether condoms were still necessary. In these accounts, trust functioned as a primary basis for sexual decision-making, with women concluding that condom use was no longer needed once they believed the relationship was monogamous and emotionally secure.

*Um, I think it was just more like being honest about what we were doing. Um, I clearly was only sleeping with him. Um, and I just had to believe him and trust him. But never during that time, um, did I say, “Oh, we should wear condoms.”*

*(37, L)*

*We had stopped usin’ condoms after I felt comfortable enough to. To stop. Um, when I felt it—I’m not sure—but when I felt it was only us in the relationship.*

*(43, AA)*

Contrasting strategy:

Some women reported using condoms even in long-term relationships, either out of caution, when the partner was a long-term casual partner, or because they didn’t feel comfortable relying solely on trust. This was often due to a personal commitment to intentional and consistent protection or a history of being hurt by partners who were not as trustworthy as they appeared.

*So, yeah, it’s been the same, basically, ’cause we just, like, I been knowin’ him over 20 years… and we do—me and him use protection [condoms]. I asked him, you know, like, “I don’t know where you been,” and we just stuck with it [using condoms]. *

*(60, AA)*

3. Reliance on HIV/STI testing

Regular testing, was often undertaken together with a partner, with or without the use of condoms, in long-term relationships. Women who stopped negotiating condoms often described a shared commitment to regular HIV/STI testing as a health maintenance strategy. However, some women expressed confidence in their partner’s health without formal HIV/STI testing, relying on assumptions about their partner’s behavior or fidelity.

*I still deal with the one person that I’ve been with for so many years, so we—and we have a monogamous relationship. So, I mean, I would hope that he’s not, you know, steppin’ out on me. So—pretty much, I have that trust, and I—I still take—I do—so for precaution, I’m always takin’ my, um, HIV testing all the time. You know, so it’s not like I don’t. So, like, I always—every three months, I take my, um, HIV testing.*

*(34, AA)*

*I mean, you know, we do engage in sexual intercourse. We usually do not use protection [condoms]—but as I said before, I do get tested frequently.*

*(34, AA)*

*Like the one man I was dating, um, when I asked him—when we started having sex and we used condoms, and I asked him if he would go get tested with me so that we could be monogamous and have unprotected sex, he just jumped right up to it. We got tested together.*

*(54, AA)*

Contrasting strategy:

In contrast, some women described continuing condom use even after both partners tested negative for HIV/STIs, framing negative results as reassuring but not, on their own, a reason to stop using condoms. Within this theme, testing functioned less as a substitute for other prevention strategies and more as an additional layer of reassurance that informed how couples calibrated protection over time.

*We using condoms. That was the thing, in terms of we would continue to use condoms since, uh, you know, results came out that it’s negative, and none of us, you know, positive with it. So we don’t need to take PrEP; we just continue to use condoms. He don’t mind.*

*(41, AA)*

#### 3.1.3. Combination Prevention

Women who employ combination prevention often used a mix of PrEP, condoms, and regular testing to maximize their protection against HIV/STIs. This strategy was particularly prevalent among women with partners living with HIV or those in relationships where there was higher perceived exposure to HIV/STIs. The combination of methods reflects an adaptive approach to protection, using multiple layers depending on the context.

1. Combination of PrEP and condoms for maximum protection

Some women employed a combination of PrEP and condoms to create a multi-layered defense against HIV/STIs. This approach was used especially in relationships where there was concern about potential exposure, such as with partners living with HIV, partners who had sex outside the relationship, or partners who injected drugs. These women valued the added assurance provided by using both methods together.

*It’s good; he’s undetectable. … Uh, we use condoms, if we want to—or the pull-out method. Yeah, some of the time. … And I’m on PrEP. *

*(31, W)*

2. Regular HIV/STI testing to supplement other methods

Women who used PrEP and/or condoms often paired these methods with regular HIV/STI testing as an additional layer of protection. Testing was seen as a safeguard to ensure that prevention strategies were working effectively, and women used it to monitor their health even if they felt secure with PrEP and condom use.

*I’ve always been somebody that uses condoms, … have always been my form of, uh—protection. Um, I also get tested very regularly and expect the same of the people that I have sex with. Um, and therefore, have had really no worries.*

*(23, W)*

3. Adapting prevention methods based on relationship context

Women who practiced combination prevention often adjusted their methods depending on the relationship. For example, they might use both PrEP and condoms with casual partners but rely on PrEP and testing in long-term relationships where they felt more secure. This flexibility allowed them to tailor their approach to different partners and situations.

*We both discussed it [using PrEP]. Both in the midst before we actually got together. Went and got our HIV tests done, and, you know, started no longer use condoms, you know.*

*(41, AA)*

Contrasting strategy:

A few women used no HIV/STI protection despite being vulnerable to exposure.

*Then I told him, “You don’t know if I have it [HIV]. We just know you have it [HIV], and if you’re gonna die, I’d rather die with you than not be with you.” So I threw out the condoms and I said, “You’re my man. I love you. We’re already married. I’m not gonna use any more condoms. That’s it.” And I was not pulling out. I wasn’t using any precautions. I threw caution to the wind.*

*(56, W)*

#### 3.1.4. PrEP as a Prevention Strategy

1. Women’s narratives about PrEP reflected both its use in higher-exposure or uncertain situations and the reasons it was often not used. Women who used PrEP typically did so when they perceived elevated HIV exposure and viewed it as an autonomous source of control and protection, often alongside condoms, HIV/STI testing, or a partner’s undetectable status. At the same time, many women perceived little need for PrEP because they trusted their partner, relied on monogamy, condoms, or testing, or faced barriers such as stigma, partner concerns, misinformation about PrEP, and limited awareness that PrEP was intended for cisgender women. PrEP use in high-exposure or uncertain situations

Women who used PrEP typically did so when they perceived heightened or uncertain HIV exposure, such as with a partner living with HIV or after unprotected sex with a partner of unknown status. In these situations, PrEP offered an added sense of control and reassurance, often alongside other prevention strategies.

*We had like a joint meeting with the doctor, and, um, I was told about it [PrEP], and then I started getting it prescribed. Well, I mean, it was huge, because, obviously, without it, you’re taking risks, …I mean, I thought it was a great idea, especially when they said that if your, you know—if your partner’s undetectable and you’re taking this medication [PrEP], the risk is very, very low.*

*(42, W)*

*Ooh, I heard about PrEP a while ago. I went in for PrEP a few years back [†]. I had, like, went out to a party, and then I ended up havin’, like, a one-night stand, and we didn’t use a condom, and I was just like, …freaked out. And he was just like, you know, you don’t have anything to worry about, but I wasn’t listenin’ to him, and I just took my ass to the hospital [to get a PEP prescription].*

*(33, L)* † Participant misidentified PEP as PrEP.

2. Perceived low need for PrEP

Some women chose not to use PrEP because they believed their risk of HIV exposure was minimal. This perception often stemmed from trust in their partner’s HIV-negative status, belief in mutual monogamy, or reliance on condoms or regular HIV/STI testing. For these women, PrEP was seen as unnecessary given their confidence in other prevention methods or the nature of their relationships.

*I knew about PrEP. I did not go on PrEP for myself. I didn’t encourage PrEP for him. … I mean, we-we were both still getting tested regularly. Um, that’s one thing that I requested of him. I said, “Listen, I don’t know what’s going on but we gotta make sure that we’re both testing.” Um, but definitely there was a lotta testing. Should I have been doing more, um, prevention? I should have, but I was more, like, testing and confirming what was being told verbally.*

*(37, L)*

*If it’s someone specific that you’re having sex with I feel like it’s a safer chance; if you don’t want to take that [PrEP], it’d be safer under those circumstances because you know your partner, your partner knows you. We know we’re not messing around with anybody else, so if we didn’t test positive before we started having sex there should be no reason [to take PrEP].*

*(62, AA)*

*I choose not to take PrEP because I feel that, yeah, there’s a lotta warning and signs that I coulda got it [HIV] or get it or whatever because of these people that I engaged with. But I think I took precaution enough.*

*(56, L)*

3. Barriers to PrEP use

A variety of barriers influenced women’s decision not to use PrEP, even when they viewed it as potentially beneficial. These barriers included concerns about stigma, misinformation about PrEP’s effectiveness or side effects, and social or cultural factors. Some women hesitated to take PrEP due to fear of being judged or due to negative associations with medication for HIV prevention, or the fear that their partner would accuse them of infidelity.

*But I have—didn’t really look into that [PrEP] or get prescribed that ’cause I’m like, “I don’t have it.” Like I’m HIV negative. I don’t need it. But I’m like—I don’t know, guess it’s just this negative stigma, like if you taking something for that, like you have it [HIV] or you just—like you’re really like out here doin’ the most right now. It’s like that negative stigma that comes with it. I don’t want that. Like I don’t want that, like, poppin’ up in my chart. Like, you know how the doctor, and people can see the medications you take and they see that, and they judge you for it like, “Oh, she’s takin’ that.” Like she—yeah, she’s out here. She’s this. She’s that.”*

*(26, L)*

*Ever since the injectable came out I’ve actually been on the fence of getting on PrEP. … I would definitely have the conversation with my husband and say, “Hey, we should both get on PrEP together.” As a Hispanic couple I know for a fact that on his end he’ll be like, “What are you doing? Why are you asking for PrEP? Are you fuckin’ other people?” Like, that—I know for a fact those will be his thought process.*

*(37, L)*

4. Misinformation and lack of awareness about PrEP

Several women hesitated to use PrEP due to misconceptions about its side effects or doubts about its efficacy. Misinformation, such as fears about long-term health impacts or misunderstandings about how PrEP works, contributed to the decision of some women not to use it. Women also described limited awareness that PrEP was intended for cisgender women and perceived that earlier public health messaging and community discourse often centered on men who have sex with men, leaving women feeling excluded from targeted outreach. This perceived exclusion had downstream implications for clinical uptake, as participants suggested it contributed to fewer conversations about PrEP in women’s health settings and less proactive prescribing by providers.

*I don’t think that, um, the way that they pitch PrEP, it’s not very appealing. Um, especially towards women ’cause they’ll say, “Oh, I have this drug you can take that prevents HIV, but you have to take it for 30 days before it kicks in. And then you hafta come back every three months to, um, get tested. But we’re not really sure of, like, the effectiveness.” [Laughter] So yeah. I-I don’t wanna be anyone’s guinea pig, kinda. … And-and it’s just, it’s just, if you, when you pitch PrEP to gay men, it works because it-it works immediately, two weeks after. So when they pitch it to them, they have everything in place, all the answers to all the questions. But when they pitch it to women, they don’t have all the answers for women.*

*(43, AA)*

*Um, yes. I-I saw the ad. I cannot remember the name of the drug, um, but I believe it was for men only. Um, I would see the commercials.*

*(36, AA)*

*I’ve not heard of [PrEP], and I actually did not know that there were, that, any type of drug like this existed, to be honest.*

*(27, L)*

#### 3.1.5. Empowerment and Negotiation in HIV Prevention

This theme captures the tension between women’s empowerment in making independent sexual health decisions and the challenges of negotiating HIV prevention methods with partners. While some women asserted autonomy in choosing prevention methods (e.g., insisting on condom use or deciding to use PrEP without involving their partner), others faced resistance from partners, making it difficult to consistently implement safer sex practices, particularly around male condom use, other methods like condoms, testing, or HIV undetectability in a partner living with HIV.

1. Autonomy in HIV prevention use decisions

Some women confidently asserted their control over condom use in their relationships, insisting on using condoms even when their partners were resistant. This sense of empowerment allowed them to prioritize their health and make decisions independently, without feeling pressured to conform to their partner’s preferences. Similarly, women who felt empowered to take control of their sexual health often made independent decisions to use PrEP, regardless of their partner’s input or awareness. This autonomy reflects a strong sense of agency, as women chose PrEP as a method of protection that did not require negotiation or consent from their partners.

*Also, I use condoms. Uh, always, I got some because when, uh, I go to the hospital, they always got like—like a bowl with some of them. It’s like a—a blue pack. So I always carry it in, in my pocket. I tell him he have to put on two. They can put two, you know.*

*(55, W)*

*I’ve always been somebody that uses condoms, … have always been my form of, uh—protection.*

*(23, W)*

*We use condoms also. Uh, like, I’m not gonna say all the time, but, uh, most of the time. Yeah, and plus, I’m usin’ my PrEP.*

*(48, AA)*

*Yes, we get them [condoms] from the clinic every time we go. And we wanted to prevent, didn’t want to get pregnant, STDs as well. So that’s when the condoms came into play. We had a lot of those to use. So, but I still felt like I wanted some more protection with him so that’s how the PrEP came about, Truvada. I got into the PrEP to get myself protected. I want to keep protecting myself. This is why I was telling him that if the shot comes out, I would like to get that shot for every two months just to have it in my system because we don’t know who’s who’s anymore. *

*(39, AA)*

2. Resistance from partners in condom negotiation

Many women faced challenges negotiating condom use with their partners, encountering resistance or reluctance that made it difficult to consistently implement safer sex practices. This resistance often stemmed from partners’ discomfort with condoms or a lack of agreement on the necessity of their use, placing women in a position where they had to navigate the use of these strategies. In some cases, women felt forced to compromise on their prevention strategies due to partner resistance, resulting in inconsistent condom use or other adjustments to their preferences, such as initiating PrEP. These negotiation struggles often led women to balance their desire for protection with maintaining the relationship, reflecting a difficult tension between personal health and relational harmony.

*We don’t use condoms. We don’t do anything as far as that. Hm-mmm. Too much. I’m puttin’ myself at risk. I’ve had HIV tested with him as well, yeah. And, um, like I said, he do too much. He got-he got two other girls that I know of. So, he want his cake, his ice cream, dessert, everything else. Hm-mmm. They want multiple women. They don’t wanna make no commitments, and they don’t wanna do anything. They don’t wanna help out. They just want sex and nothin’ else but that.*

*(56, AA)*

*Condom use? We don’t—we don’t use condoms. How did that decision come? His preference. It was his preference. I had second thoughts. I be goin’ to the doctor and makin’ sure, gettin’ my blood and everything tested and makin’ sure everything is all right.*

*(31, AA)*

3. Partner control and challenges to empowerment

Some women encountered situations in which their partners exercised control over sexual health decisions, making it difficult for them to assert their own preferences or derive a joint agreement. In these cases, partners might outright refuse condoms, pressure the woman to forego certain prevention methods, or downplay the importance of safer sex practices, thereby undermining the woman’s ability to make empowered decisions.

*Um, uh, unfortunately, on the evening that I’m referring to, a condom was not used, and that was not a choice of my own.*

*(23, W)*

*Um, I’ve tried [talking to my husband about using condoms]. And that did not go over well. It led to an argument. So I just left it alone. Basically, [he said] that I’m his wife. So there’s no need to. Yeah, ’cause again, those are, like, my concerns. So I’m just—like again back to something like I don’t know if you’re, like, on the up and up. You probably don’t trust me and think I’m on the up and up. So it’s like I was kind of doing that—more for myself ’cause I didn’t trust him at the time. So I was like, “Can we just do that, but, like, can you wrap it?” And he—it did not go over well.*

*(26, L)*

*We didn’t use condoms. We were—we lived together. And he won’t use it if—like I told him, you want some sex, you gotta put on a condom, and then he would get mad ‘cause he don’t wanna use a condom.*

*(56, AA)*

4. Communication about prevention as an expression of empowerment

Some women described directly initiating conversations with partners about condom use, HIV testing, or other prevention strategies. These exchanges illustrated how women asserted their preferences, raised concerns about sexual health, and sought greater clarity about risk within the relationship.

*I don’t think it [not using condoms and HIV testing] was a decision that we both came to together. I mean, I feel like for the most part, I’m in control of my own health, so I do whatever I feel is best for me. Uh, we did recently have a conversation about it, and I told him maybe he should go get tested.*

*(34, AA)*

*I was like, “Hey—I don’t know really what you’re doin’. Um, I’m clearly havin’ unprotected sex with you. Like, I need to know what’s goin’ on.” Um, so then he said, “Oh, I’m—I don’t care. I’ll go get tested. Like, I’m not doin’ anything.” Um, so yeah, it was me. I brought up like we need to test. … And of course he’s like, “Are you-are you with other people? Are you cheating? Are you—“ “No. I’m just sayin’ I don’t know what you’re doing. You don’t know what I’m doing. Let’s just do this.”*

*(37, L)*

5. Discreet prevention methods as empowerment

Women who struggled with negotiating condom use or other methods often turned to discreet or autonomous forms of protection, such as PrEP, as a way to maintain control over their sexual health without needing to involve their partner in the decision. This allowed them to circumvent the negotiation process while still protecting themselves.

*Have I told my partner [I’m on PrEP]? Well, I never told him that I took—that I was takin’ it [PrEP]. I just felt like—like, I’m puttin’ this inside my body, that is my body. So I feel like I’m gonna have to go through all this headache where [he says], “Oh, why you doin’ that?”*

*(34, AA)*

#### 3.1.6. Abstinence as a Strategy for HIV/STI Prevention

For some women, abstinence was a deliberate strategy to avoid HIV/STI exposure. Whether it was a temporary decision during uncertain times or a more permanent lifestyle choice, abstinence allowed women to maintain control over their sexual health by removing the risks associated with sexual activity. This theme emphasizes how abstinence is often viewed as a form of self-empowerment.

1. Temporary abstinence during uncertainty

Some women chose abstinence as a temporary strategy during periods of uncertainty or transitions in their lives, such as when they were between partners or after ending a relationship where they felt vulnerable. This temporary decision allowed them to protect themselves while they reassessed their sexual health and circumstances.

*Well, I’m dating now I just started this year. So we’re gonna see how it goes. I don’t have any protection, but I’m not having sex at all neither. But this is what we’ve been talking about maybe five weeks. I want to take that as slow as possible.*

*(39, AA)*

*I’m-I’m abstinent. I don’t have anyone in my life. I don’t get high. I’m not at risk. If I-if I was to do anything, it might be sleep with someone who might have it and not tell me, but if I do that, I’ll use protection. Or you know, yeah, condoms.*

*(56, AA)*

2. Long-term abstinence as a lifestyle choice

A few women described choosing abstinence as a long-term strategy. This decision was often framed as a lifestyle choice that allowed them to maintain full control over their sexual health. For these women, abstinence was less about temporary protection and more about asserting autonomy over their bodies and relationships. Choosing not to engage in sexual activity allowed them to feel in control of their health and well-being, particularly in situations where they felt vulnerable or unsure about potential partners.

*I’m always careful how I, um, consume myself in these situations. Um, that’s why I take the long gaps [from having sex], and that’s why I-I avoid having sex, um, most of the time. I have to really feel it in my system. And, you know, I could be without for a while.*

*(34, AA)*

#### 3.1.7. Effects of the COVID-19 Pandemic on Sexual Behavior and HIV/STI Prevention

1. Women reporting no effect of the pandemic

About half of the women interviewed stated that the COVID-19 pandemic did not affect their sexual behavior or HIV/STI prevention practices because their sexual activity was embedded in steady or familiar relationships and day-to-day routines that continued through the pandemic. This often involved marital or primary partnerships but was also evident among women who described open relationships or multiple partners and who emphasized that they continued seeing the “same people” and maintained their usual patterns.

*I wasn’t worried about it [COVID] at all… Cause you’re home anyway and it didn’t matter. *

*(31, W)*

*Nope… It [COVID] didn’t involve my sex life at all.*

*(46, AA)*

*I still see the same people I see… I don’t think it’s [COVID] affected my sex life.*

*(26, W)*

2. Women reporting an effect of the pandemic
  Among women who reported that the COVID-19 pandemic affected their sexual behavior and/or HIV/STI prevention practices, the most common impacts reflected (1) reduced opportunities for meeting or being physically close to partners, (2) a shift in prevention conversations toward COVID exposure risk, and (3) broader psychosocial and relationship strain that reduced sexual interest or complicated relationship stability.
  2.1. Reduced opportunity for sexual contact and new partner formation
    Many women described pausing dating and sexual activity due to quarantine, social distancing, and reduced social opportunities, resulting in fewer (or no) partners. As one participant summarized, “I just have been staying away from people…keeping to myself… [my relationships were] nonexistent basically.” (21, L) Others described simply being “in the house most of the time” and “just…weren’t havin’ sex” because “nobody was goin’ outside.” (31, AA) For some, the disruption was framed as fewer opportunities to meet partners who felt worth pursuing: “it added a new layer to dating… [and] you meet less people that you actually want to have sex with.” (23, W) “I mean, there is no more casual sex… Now you gotta worry about if you breathe in something, you gonna die… So you can’t find other partners.” (54, AA)
  2.2. COVID-focused partner screening and altered prevention priorities
    Across accounts, sexual decision-making became shaped by COVID-19 exposure and infection risk, sometimes eclipsing HIV/STI prevention concerns: “I’m just scared [of COVID]. I don’t even want to be touched.” (47, L) Women described using quarantine, vaccination status, and COVID testing as informal “gatekeeping” for intimacy: “Did you get your shots?…I can’t see you for 14 days…let me take…a home swab before we get together.’ “(41, W) In one case, prevention shifted from condoms and HIV/STI concerns to COVID exposure surveillance: “It was definitely a lot of like, ‘Who you been around? Where you been at?’ Not like, ‘Are you wearing protection [condoms]?’ Those were not the questions for sure.” (37, L) Women also described adaptive substitutions for physical intimacy, including solo sexuality, such as “I never in my life thought I would go to Amazon and buy me a toy, but I definitely did. I, um—it was definitely something that I picked up during COVID. I-I learned how to self-masturbate.” (37, L)
  2.3. Psychosocial distress and relationship strain shaping sexual desire and stability
    For some women, pandemic stressors intensified depression, grief, and relational instability, diminishing sexual interest and straining relationships. One participant described entering “isolation mode” after a major loss: “I went into, like, a real bad depression, so I didn’t wanna be bothered with anybody. I didn’t wanna like—men were, like, the least of my concerns.” (33, L) Others connected decreased sexual desire to depression or relational conflict: “being depressed would make you not wanna do [sexual] things… [the relationship was] troubled…when there’s no sex in the relationship it can put a strain.” (34, AA) For some couples, forced proximity due to quarantine amplified pre-existing problems, highlighting how the pandemic reshaped not only sexual behavior but relationship trajectories: “a little bit before COVID we started having marriage issues, but then with COVID being home a lot more together, um, you notice a lot of things that you don’t get to notice when you’re kind of like always just, like, crossin’ one another, like short interactions. Um, so actually our sex life kind of died. It really did unfortunately… We’re actually getting a divorce.” (27, L)

### 3.2. Comparative Analysis: Differences in HIV/STI Prevention Strategies by Race/Ethnicity, Site Location, and Age

#### 3.2.1. Differences by Race and Ethnicity

Across all racial/ethnic groups, partner type in tandem with relationship features played a central role in shaping HIV/STI prevention strategies; however, the way in which women adapted these strategies varied. Black and Latina women were more likely to adjust their approaches based on their partner’s health status, behavior, or past, whereas White women tended to assume monogamy in their primary relationships, leading to a greater reliance on trust. Latina women were particularly inclined to discontinue condom use with primary partners, trusting in the relationship’s stability and mutual HIV/STI testing. In contrast, Black women were more cautious, often maintaining condom use and regular testing, even in committed relationships.

While all groups used testing as a key prevention strategy, White women were more likely to engage in combination prevention, pairing PrEP and condoms to manage risk, particularly in perceived high-risk situations or relationships with HIV-positive partners. Black women were the least likely to use PrEP consistently, but they demonstrated a strong sense of agency through using a range of prevention strategies, like selective condom negotiation, regular HIV/STI testing, or abstinence. Although condom negotiation is used by Black women as a prevention strategy, some expressed negative attitudes due to discomfort. Latina women, though often more open to PrEP, faced substantial barriers to accessing and using it, such as concerns about stigma and side effects.

In terms of empowerment, White women showed the most autonomy in making prevention decisions and encountered the least resistance from partners regarding condom use. Black women often faced greater challenges negotiating prevention behaviors but asserted control through other means, such as testing or situational condom use. Abstinence was most frequently reported by Black women as an intentional strategy, providing them with a sense of empowerment and control over their health, while Latina women were the least likely to use abstinence, preferring other prevention and risk-management strategies.

Across all groups, personal empowerment, partner dynamics, and cultural context significantly shaped women’s approaches to HIV/STI prevention, with each group reflecting distinct adaptations to these shared challenges.

#### 3.2.2. Differences by Site Location

Across both NYC and Rochester, women employed similar HIV/STI prevention strategies, such as relying on trust, routine HIV/STI testing, and selective condom and PrEP use, influenced by relationship dynamics and partner type. However, key differences emerged in how prevention strategies were implemented. Women in NYC were more likely to integrate PrEP into their prevention plans, particularly in high-risk situations or with HIV-positive partners, often employing combination prevention by integrating PrEP with condom use and testing. In contrast, Rochester women were more inclined to rely on their partners’ perceived characteristics, behaviors, past history, and testing with discontinued condom use and less reliance on PrEP.

In both locations, challenges around negotiating condom use with partners were evident. NYC women sometimes used PrEP to navigate these challenges, particularly when partners resisted condom use, whereas women in Rochester faced more obstacles in asserting condom use, often resulting in reliance on trust or testing as alternatives. These differences reflect how partners influence prevention behaviors, with Rochester women appearing more influenced by traditional relationship norms and stigma, whereas women in NYC took a more active role in managing their sexual health.

Abstinence was a more prominent strategy among Rochester women, who often framed it as a deliberate choice, especially following negative relationship experiences. Women in NYC, on the other hand, more often described intentional prevention and risk-management strategies such as PrEP and condoms, focusing on maintaining control in relationships with varied perceived levels of HIV/STI exposure.

#### 3.2.3. Generational Differences

Across all age groups, partner type was a significant factor in guiding HIV/STI prevention strategies, though the emphasis and methods varied by age. Younger women (35 and under) often relied on trust and monogamy to phase out condom use in primary relationships, with the assumption that commitment alone offered protection. This group was more likely to rely on HIV/STI testing as a regular risk-management practice and displayed fewer behaviors around maintaining condom use over time. In contrast, middle-aged women (age 36–54) frequently incorporated HIV/STI testing as a supplement to condom use, particularly in primary relationships, balancing trust with ongoing verification of partner health status as a calculated health assessment. Older women (55 and above) showed the most consistent condom use with primary partners, demonstrating a cautious approach that relied less on trust alone and more on the routine use of prevention methods even in long-term relationships.

In terms of combination prevention strategies, middle-aged women tended to incorporate PrEP into their prevention strategies, particularly in high-risk situations, reflecting a greater openness to combination prevention methods compared to the other age groups. They also included regular HIV/STI testing as an important aspect of their approach, using it in conjunction with other strategies to ensure protection. Younger and older women, by contrast, tended to adopt PrEP less frequently. Younger women often preferred to focus on partner characteristics to manage perceived risks rather than relying on PrEP or combination prevention.

Across age groups, women exhibited varying levels of empowerment and autonomy in their prevention choices, with notable differences in how they navigated partner resistance, especially to condom use. Younger women tended to express independence in decision-making, frequently taking control over prevention strategies despite encountering the highest levels of partner resistance to condom use. This resistance often led younger women to balance active negotiation with a reliance on trust and monogamy, especially in committed relationships where they prioritized relationship dynamics over consistent condom use. In contrast, middle-aged women faced less partner resistance, allowing them to navigate prevention more smoothly; they often used HIV/STI testing and open discussions as cooperative tools to maintain control without the need for frequent negotiation. Middle-aged women’s approach was pragmatic, blending autonomy with a degree of relational ease in their prevention strategies. Older women, however, demonstrated a more cautious approach that largely bypassed negotiation challenges, as they relied on consistent condom use or abstinence as intentional strategies. By choosing abstinence or routine condom use, older women minimized the need for negotiation with partners, often framing these choices as a form of self-empowerment and control. Across all groups, empowerment and autonomy in prevention were shaped by both generational norms and partner dynamics, with each age group adapting strategies according to the degree of partner cooperation they expected or encountered.

Lastly, abstinence emerged as a notable strategy among older women, who used it more frequently as a form of empowerment and control over their sexual health, especially following negative experiences or during periods of emotional recovery. In contrast, younger women rarely used abstinence as a prevention strategy, while middle-aged women mentioned it situationally, often as a temporary choice during uncertain times.

This age-based analysis highlights distinct approaches to HIV/STI prevention, with younger women prioritizing trust and partner type, middle-aged women relying on HIV/STI testing as a core strategy, and older women favoring consistent condom use and abstinence for greater control and security.

## 4. Discussion

This analysis of cisgender women who are PrEP-eligible highlights their prevention ecologies—relationship- and context-dependent combinations of strategies that shift with partner dynamics, access constraints, and life circumstances. Empowerment was reflected not as an abstract trait, but as women’s practical ability to assess risk and to initiate, negotiate, or enact prevention strategies under varying relationship and structural conditions. Women’s narratives closely reflected the “condoms to trust” transition: condom use was most common early in relationships and with casual partners and often declined as trust and perceived monogamy increased [27,39,40,41,42]. Because unmet monogamy expectations can increase HIV/STI vulnerability [43,44,45], women described compensating by adding periodic HIV/STI testing (sometimes jointly) and/or PrEP during heightened uncertainty [20,43,46,47,48,49]. Some also relied on partial risk-reduction practices (e.g., partner disclosure, perceived health-seeking behavior, withdrawal, avoiding certain sex acts), consistent with prior accounts when options are constrained [43]. Notably, PEP did not emerge as a salient prevention strategy in participants’ narratives. Only about half of the women had ever heard of PEP, most of those who had heard of it knew little about it, and only two women reported ever having been prescribed it. This absence is itself meaningful, suggesting that event-driven biomedical prevention may remain underrecognized or underexplained for cisgender women. Together, these narratives suggest that prevention counseling that assumes stable behaviors may miss the shifting prevention ecologies women employ that are context-dependent. These ecologies also varied by race/ethnicity, site, and age and were shaped by gender-power dynamics and COVID-19 disruptions.

PrEP use in this sample mirrored broader U.S. patterns of low uptake and persistence among cisgender women despite substantial interest [15,49]. Women often framed PrEP as episodic “insurance” in HIV-serodifferent relationships or when condom negotiation was difficult, but few used it consistently and some discontinued when relationships stabilized or perceived susceptibility declined [50,51,52,53]. PrEP was described as reducing anxiety and increasing control among women in HIV-serodifferent relationships or when condom negotiation was difficult [48,54,55]. Conversely, low perceived susceptibility (rooted in trust or reliance on testing) reduced interest and underscored misalignment between algorithm-based eligibility and women’s own risk appraisals [15,56]. The COVID-19 pandemic intensified these dynamics: women described clinic closures, reduced in-person testing and PrEP access, and greater reliance on telehealth and home testing, consistent with broader reports of pandemic-related service disruptions [57,58].

In addition, our findings advance prior literature by comparing prevention narratives across racial/ethnic groups, age, and location. Prior work has shown that Black and Latina women face disproportionate structural and interpersonal barriers to prevention, including racism, sexism, stigma, and limited access to PrEP and sexual health services [17,24,59]. Black women in our study often reported heightened concern about HIV, and were more likely to maintain condom use, routine HIV testing, and intentional abstinence [60]. Yet, they reported frequent partner resistance to condoms and testing, consistent with studies among Black women in the U.S. South [61,62]. Latina women more often framed stopping condoms with primary partners as a sign of relationship stability and mutual testing and expressed openness to PrEP [63,64] while voicing strong concerns about stigma, side effects, family or partner disapproval, aligning with work on Hispanic women’s PrEP perceptions [63,65,66,67,68].

Our age-stratified findings align with prior research showing that condom use commonly declines as relationships become more intimate, and that younger women often interpret commitment, monogamy, and partner trust as indicators of reduced HIV/STI risk [69]. In this context, younger women’s greater reliance on HIV/STI testing as a prevention strategy is consistent with evidence that relationship characteristics and preventive health consciousness motivate testing decisions among young women [69]. Midlife women’s tendency to rely on ongoing HIV/STI testing—even within primary partnerships—extends the literature describing pragmatic risk management that balances relational trust with active verification [70]. Our finding that women in this middle age group reported greater integration of PrEP is inconsistent with prior studies indicating no differences in awareness, attitudes, or intention to initiate PrEP across age groups [71]. In our study, older women tended to report more consistent condom use, which contrasts with some prior studies suggesting that condom use is often infrequent among midlife and older women and that perceived HIV risk can be low in later life [72,73]. For some women, aging may also produce a “protective shift” in prevention practices—potentially driven by accumulated relationship experiences and a stronger preference for strategies that minimize negotiation burden—while reinforcing that abstinence and fidelity-oriented approaches may be framed as self-protective ideals within prevention decision-making.

Women in New York City were more likely than those in Rochester to include PrEP in their prevention strategies and to describe it as a response to partner resistance to condoms, mirroring evidence that PrEP resources cluster in large urban centers [74,75]. Rochester participants more often relied on perceived partner characteristics, testing, and abstinence and described fewer opportunities to access PrEP, consistent with policy analyses of geographic inequities [18].

These findings have several implications for HIV prevention guidelines and practice. First, guidelines and clinical practice should explicitly recognize that women use evolving prevention strategies. Counseling frameworks that help women map partner types, relationship histories, and prevention goals and then assemble layered strategies—combining condoms, PrEP/PEP, regular HIV/STI testing, and, when appropriate, abstinence—may be more acceptable and effective than single-method messages [76,77]. Second, because many women rely on testing as a substitute for or complement to condoms and PrEP, status-neutral models of care should ensure easy access to HIV/STI testing (including self-testing kits) across primary care, family planning, obstetrics/gynecology, and community venues, with clear pathways to PrEP and other services [78,79,80,81]. Third, given the centrality of partner dynamics, routine assessment of intimate partner violence, coercion, and partner refusal of condoms or testing should be incorporated into prevention guidelines, alongside linkage to trauma-informed intimate partner violence services and discreet options such as PrEP and self-testing [77,82,83,84]. Finally, interventions must be culturally and contextually tailored, addressing structural barriers, medical mistrust, and anticipated stigma among Black and Latina women through strategies such as community-based programs, peer navigation, mobile apps, and social-media [64,85,86,87,88].

Strengths of this study include its focus on PrEP-eligible cisgender women in two distinct geographic settings, attention to racial and ethnic identity, and generational differences, and use of a narrative, framework-guided approach. Limitations include recruitment from two New York State sites; accordingly, the findings should be interpreted in relation to these specific contexts and may not transfer fully to other settings or populations. Reliance on cross-sectional interviews requiring retrospective reconstruction of trajectories and possible social desirability bias are additional limitations. However, contextual differences were apparent and provide insights on regional considerations that highlight the need for robust state-wide initiatives.

Future research should use longitudinal mixed methods designs to track how women’s prevention strategies evolve across relationship transitions. Implementation studies are needed to test women-centered counseling models that explicitly support layered context-sensitive strategies and to examine how policy changes—such as Medicaid expansion, pharmacist prescribing of PrEP, and large-scale distribution of self-tests—are experienced by women in diverse racial/ethnic and geographic groups [18,81,86,89,90,91]. There is particular need for studies focused on Latina women, older women, and women in smaller cities and rural areas, using more explicit intersectional analytic approaches. Finally, research that integrates women’s narratives with partners’ and providers’ perspectives to understand how relationship and system dynamics shape prevention choices are needed [67,76,92,93,94].

## 5. Conclusions

In this qualitative analysis of 48 PrEP-eligible cisgender women in New York City and Rochester, NY, women’s HIV/STI prevention strategies were described as evolving prevention ecologies shaped by relationship stage, partner dynamics, race/ethnicity, age, and COVID-19 disruptions. Although limited to two sites and interview recall, women often moved from condoms to trust and testing as commitment increased, using PrEP episodically during uncertainty or when condom negotiation was difficult; some used abstinence deliberately, and PEP was largely absent from women’s prevention ecologies. Findings support women-centered, culturally and contextually tailored, status-neutral services that expand routinized HIV/STI testing (including self-testing) and access to PrEP and PEP. Longitudinal implementation studies should test scalable counseling models and reduce geographic inequities.

## Figures and Tables

**Table 1:** Sample characteristics (*N* = 47 ^‡^, unless otherwise indicated due to missing data).

Characteristic	Descriptive Statistic
Age (years) ^†^	41 (19–63)
Race	
Black or African American	24 (51.1)
White	10 (21.3)
Asian	1 (2.1)
Other	12 (25.5)
Hispanic/Latina	14 (29.8)
Education	
Did not complete high school	8 (17.0)
Complete high school	13 (27.7)
Post-secondary education	21 (44.7)
Master’s degree or higher	4 (8.5)
Employment Status	
Employed or Retired	27 (57.4)
Unemployed/Assistance	18 (38.3)
Student/Other	2 (4.3)
Site	
New York City, NY	29 (61.7)
Rochester, NY	18 (39.3)
Sexual Relationship, PrEP, and Substance Use Characteristics	
Have current primary sex partner	31 (66.0)
Current primary sex partner is living with HIV	9/31 (29.0)
One or more casual sex partners last 3 months	25/45 (55.6)
Number of casual partners last 3 months ^†^	1 (1–9)
Had primary and casual sex partners last 3 months	13/45 (28.9)
Exchanged sex for drugs or money last 3 months	8/46 (17.4)
Currently prescribed PrEP	12 (25.5)
Used non-injection illicit drugs last 3 months	9/46 (19.6)
Injected drugs last 3 months	0 (0.0)
Ever injected drugs	2/46 (4.3)

^†^ Median (range). ^‡^ 1 qualitative participant did not complete the quantitative survey.

## Data Availability

Raw qualitative interview transcripts cannot be publicly shared due to assurances of confidentiality provided to our participants as per the informed consent agreement, and the possibility of individual identification based on information revealed in the interviews.

## Abbreviations

The following abbreviations are used in this manuscript:

AA	African American
CDC	Centers for Disease Control and Prevention
COVID	Coronavirus Disease
HIPAA	Health Insurance Portability and Accountability Act
HIV	Human Immunodeficiency Virus
IPV	Intimate Partner Violence
L	Latina
NY	New York
NYC	New York City
PEP	Post-Exposure Prophylaxis
PrEP	Pre-Exposure Prophylaxis
QDS	Questionnaire Development System
SARS-CoV-2	Severe Acute Respiratory Syndrome Coronavirus 2
SAS	Statistical Analysis System
STI	Sexually Transmitted Infection
W	White
WISE	Women’s Study in Sexual Health and Empowerment

## References

[1] Centers for Disease Control and Prevention (2024). HIV Surveillance Supplemental Report: Estimated HIV Incidence and Prevalence in the United States, 2018–2022.

[2] Martin E., Ansari B., Hart-Malloy R., Smith D., Delaney K., Gift T., Berruti A.A., Trigg M. (2021). Racial and ethnic disparities in HIV diagnoses among heterosexually active persons in the United States nationally and by state, 2018. PLoS ONE.

[3] Rimmler S., Golin C., Coleman J., Welgus H., Shaughnessy S., Taraskiewicz L., Lightfoot A.F., Randolph S.D., Riggins L. (2022). Structural barriers to HIV prevention and services: Perspectives of African American women in low-income communities. Health Educ. Behav..

[4] Aholou T., Murray A., Sutton M., Wright E.R., Carnes N. (2016). The social, structural, and clinical context of HIV prevention and care for Black/African American and Hispanic women/Latinas in the United States. Understanding the HIV/AIDS Epidemic in the United States: The Role of Syndemics in Shaping the Public’s Health.

[5] Reaves T., Lewis R., Dasgupta S., Lyons S.J., Tie Y., Nair P., Carree T., Hu X., Raiford J.L., Marcus R. (2024). The use of HIV prevention strategies and services reported by Black women with a risk for and with HIV in the United States. AIDS Behav..

[6] Crosby R., Yarber W., Meyerson B. (2000). Prevention strategies other than male condoms employed by low-income women to prevent HIV infection. Public Health Nurs..

[7] Osakwe C., Van Der Drift I., Opper C., Zule W., Browne F., Wechsberg W. (2023). Condom use at last sex and sexual negotiation among young African American women in North Carolina: Context or personal agency. J. Racial Ethn. Health Disparities.

[8] Teitelman A., Calhoun J., Duncan R., Washio Y., McDougal R. (2015). Young women’s views on testing for sexually transmitted infections and HIV as a risk reduction strategy in mutual and choice-restricted relationships. Appl. Nurs. Res..

[9] Katz B.P., Fortenberry J.D., Zimet G.D., Blythe M.J., Orr D.P. (2000). Partner-specific relationship characteristics and condom use among young people with sexually transmitted diseases. J. Sex Res..

[10] Higgins J.A., Hoffman S., Dworkin S.L. (2010). Rethinking gender, heterosexual men, and women’s vulnerability to HIV/AIDS. Am. J. Public Health.

[11] Bond K.T., Chandler R., Chapman-Lambert C., Jemmott L.S., Lanier Y., Cao J., Nikpour J., Randolph S.D. (2021). Applying a nursing perspective to address the challenges experienced by cisgender women in the HIV status neutral care continuum: A review of the literature. J. Assoc. Nurses AIDS Care.

[12] Siegler A., Mouhanna F., Giler R.M., Weiss K., Pembleton E., Guest J., Jones J., Castel A., Yeung H., Kramer M. (2018). The prevalence of pre-exposure prophylaxis use and the pre-exposure prophylaxis-to-need ratio in the fourth quarter of 2017, United States. Ann. Epidemiol..

[13] Bradley E., Forsberg K., Betts J., DeLuca J., Kamitani E., Porter S., Sipe T.A., Hoover K.W. (2019). Factors affecting pre-exposure prophylaxis implementation for women in the United States: A systematic review. J. Women’s Health.

[14] Allen D.C., Rabionet S.E., Popovici I., Zorrilla C.D. (2022). Acknowledging and addressing the gender disparity in pre-exposure prophylaxis use for HIV prevention. J. Pharm. Health Serv. Res..

[15] Teitelman A.M., Tieu H.V., Flores D., Bannon J., Brawner B.M., Davis A., Gugerty P., Koblin B. (2022). Individual, social and structural factors influencing PrEP uptake among cisgender women: A theory-informed elicitation study. AIDS Care.

[16] Vora N., Badowski M.E. (2024). HIV preexposure prophylaxis and postexposure prophylaxis in women: A comprehensive guide for healthcare providers. Ther. Adv. Infect. Dis..

[17] Boudreaux J., Valdebenito C.M., Pichon L.C. (2025). Identifying access barriers to PrEP among cisgender Black/African American women in the United States: A systematic review of the literature. Healthcare.

[18] Chory A., Bond K. (2024). Access to PrEP and other sexual health services for cisgender women in the United States: A review of state policy and medicaid expansion. Front. Public Health.

[19] Britton O., Hull S., Xu M., Scott R. (2025). Identifying provider-level barriers to provision of PrEP services for cisgender women: Application of the disclosure decision-making model. J. Health Commun..

[20] Baldwin A., Light B., Allison W. (2021). Pre-exposure prophylaxis (PrEP) for HIV infection in cisgender and transgender women in the U.S.: A narrative review of the literature. Arch. Sex. Behav..

[21] Haynes A.S., Markham C., Schick V., Suchting R., Parthasarathy N., Choudhury S., Hill M.J. (2024). A systematic review and narrative synthesis of factors affecting pre-exposure prophylaxis willingness among Black women for HIV prevention. AIDS Behav..

[22] Hill L., Allison O., Adeniran O., Jones M., Ayangeakaa S., Stancil T., Phillips-Weiner K.J., Lightfoot A.F., McKellar M.S., Golin C.E. (2025). PrEP knowledge and perceptions among women living in North Carolina public housing communities. PLoS ONE.

[23] Randolph S., Golin C., Welgus H., Lightfoot A., Harding C., Riggins L. (2020). How perceived structural racism and discrimination and medical mistrust in the health system influences participation in HIV health services for Black women living in the United States south: A qualitative, descriptive study. J. Assoc. Nurses AIDS Care.

[24] Pratt M., Jeffcoat S., Hill S., Gill E., Elopre L., Simpson T., Lanzi R., Matthews L.T. (2022). “We feel like everybody’s going to judge us”: Black adolescent girls’ and young women’s perspectives on barriers to and opportunities for improving sexual health care, including PrEP, in the southern U.S. J. Int. Assoc. Provid. AIDS Care.

[25] Anderson K.M., Blumenthal J., Jain S., Sun X., Amico K., Landovitz R., Burke J.G., Javanbakht M., Gorbach P.M. (2024). The impact of intimate partner violence on PrEP adherence among U.S. cisgender women at risk for HIV. BMC Public Health.

[26] El-Bassel N., Wechsberg W.M. (2012). Couple-based behavioral HIV interventions: Placing HIV risk-reduction responsibility and agency on the female and male dyad. Couple Fam. Psychol. Res. Pract..

[27] Godinho C., Pereira C., Pegado A., Luz R., Alvarez M.-J. (2024). Condom use across casual and committed relationships: The role of relationship characteristics. PLoS ONE.

[28] Krakower D., Solleveld P., Levine K., Mayer K. (2020). Impact of COVID-19 on HIV preexposure prophylaxis care at a Boston community health center. J. Int. AIDS Soc..

[29] DiNenno E.A. (2022). HIV testing before and during the COVID-19 pandemic—United States, 2019–2020. Morb. Mortal. Wkly. Rep..

[30] Huang Y.L.A., Zhu W., Wiener J., Kourtis A.P., Hall H.I., Hoover K.W. (2022). Impact of coronavirus disease 2019 (COVID-19) on human immunodeficiency virus (HIV) pre-exposure prophylaxis prescriptions in the United States—A time-series analysis. Clin. Infect. Dis..

[31] Conley C., Johnson R., Bond K., Brem S., Salas J., Randolph S. (2022). US Black cisgender women and pre-exposure prophylaxis for human immunodeficiency virus prevention: A scoping review. Women’s Health.

[32] Taggart T., Milburn N.G., Nyhan K., Ritchwood T.D. (2020). Utilizing a life course approach to examine HIV risk for Black adolescent girls and young adult women in the United States: A systematic review of recent literature. Ethn. Dis..

[33] Gelberg L., Andersen R.M., Leake B.D. (2000). The behavioral model for vulnerable populations: Application to medical care use and outcomes for homeless people. Health Serv. Res..

[34] Opara I., Martin R., Hill A., Calhoun A. (2023). Addressing gendered racism against Black girls using a strengths-based empowerment-intersectional framework for sexual health and substance use prevention programming. Health Promot. Pract..

[35] Hull G.T., Scott P.B., Smith B., Combahee River Collective (1982). A Black Feminist Statement. But Some of Us Are Brave.

[36] Crenshaw K. (1989). Demarginalizing the Intersection of Race and Sex: A Black Feminist Critique of Antidiscrimination Doctrine, Feminist Theory and Antiracist Politics. Univ. Chic. Leg. Forum.

[37] Collins P.H. (2022). Black Feminist Thought, Knowledge, Consciousness, and the Politics of Empowerment.

[38] Furber C. (2010). Framework analysis: A method for analysing qualitative data. Afr. J. Midwifery Women’s Health.

[39] Corbett A.M., Dickson-Gomez J., Hilario H., Weeks M.R. (2009). A little thing called love: Condom use in high-risk primary heterosexual relationships. Perspect. Sex. Reprod. Health.

[40] Norris A., Carey K., Guthrie K., Rich C., Krieger N., Kaplan C., Carey M.P. (2020). Partner type and young women’s sexual behavior: A qualitative inquiry. Archiv Sex. Behav..

[41] Macaluso M., Demand M.J., Artz L.M., Hook E.W. (2000). Partner type and condom use. AIDS.

[42] Duncan E.A.W. (2011). Influence of partner type on condom use. J. Hum. Behav. Soc. Environ..

[43] Hotton A.L., French A.L., Hosek S.G., Kendrick S.R., Lemos D., Brothers J., Kincaid S.L., Mehta S.D. (2015). Relationship dynamics and sexual risk reduction strategies among heterosexual young adults: A qualitative study of sexually transmitted infection clinic attendees at an urban Chicago health center. AIDS Patient Care STDs.

[44] Lehmiller J. (2015). A comparison of sexual health history and practices among monogamous and consensually nonmonogamous sexual partners. J. Sex. Med..

[45] Ibañez G.E., Whitt E., Avent T., Martin S.S., Varga L.M., Cano M.A., O’Connell D.J. (2017). ‘Love and trust, you can be blinded’: HIV risk within relationships among Latina women in Miami, Florida. Ethn. Health.

[46] Blumenthal J., Landovitz R., Jain S., He F., Kofron R., Ellorin E., Ntim G.M., Stockman J.K. (2021). Pre-exposure prophylaxis perspectives, sociodemographic characteristics, and HIV risk profiles of cisgender women seeking and initiating PrEP in a US demonstration project. AIDS Patient Care STDs.

[47] Teitelman A.M., Chittamuru D., Koblin B.A., Davis A., Brawner B.M., Fiore D., Broomes T., Ortiz G., Lucy D., Tieu H.-V. (2020). Beliefs associated with intention to use PrEP among cisgender U.S. women at elevated HIV risk. Arch. Sex. Behav..

[48] Willie T.C., Monger M., Nunn A., Kershaw T., Stockman J.K., Mayer K.H., Chan P.A., Adimora A.A., Mena L.A., Knight D. (2021). “PrEP’s just to secure you like insurance”: A qualitative study on HIV pre-exposure prophylaxis (PrEP) adherence and retention among black cisgender women in Mississippi. BMC Inf. Dis..

[49] Blackstock O.J., Platt J., Golub S.A., Anakaraonye A.R., Norton B.L., Walters S.M., Sevelius J.M., Cunningham C.O. (2021). A pilot study to evaluate a novel pre-exposure prophylaxis peer outreach and navigation intervention for women at high risk for HIV infection. AIDS Behav..

[50] Blumenthal J., Jain S., He F., Amico K.R., Kofron R., Ellorin E., Stockman J.K., Psaros C., Ntim G.M., Chow K. (2021). Results from a PrEP demonstration project for at-risk cisgender women in the United States. Clin. Infect. Dis..

[51] Pyra M., Johnson A.K., Devlin S., Uvin A.Z., Irby S., Stewart E., Blum C., Green M., Haider S., Hirschhorn L.R. (2022). HIV pre-exposure prophylaxis use and persistence among black ciswomen: “Women need to protect themselves, period”. J. Racial Ethn. Health Disparities.

[52] Pyra M., Brewer R., Rusie L., Kline J., Willis I., Schneider J. (2022). Long-term HIV pre-exposure prophylaxis trajectories among racial & ethnic minority patients: Short, declining, & sustained adherence. J. Acquir. Immune Defic. Syndr..

[53] McMahon J.M., Simmons J., Braksmajer A., LeBlanc N. (2022). HIV-serodifferent couples’ perspectives and practices regarding HIV prevention strategies: A mixed methods study. PLoS Glob. Public Health.

[54] Knight D., Monger M., Phillips K., Antar A., Baral S., Stockman J., Nunn A., Chan P., Mayer K., Mena L. (2024). PrEP initiation and adherence among Black cisgender women in Mississippi: The role of HIV and PrEP stigma and social support. Women’s Health.

[55] Phuntsog T.D., Stockman J.K., Amico K.R., Kofron R., Morris S., Landovitz R.J., Strathdee S., Moore D.J., Blumenthal J. (2026). PrEP as a “hidden message”: The impact of partner dynamics on oral PrEP adherence among cisgender women in serodiscordant relationships. AIDS Behav..

[56] Shankaran S., Friedman E., Devlin S., Kishen E., Mason J., Sha B., Payne D., Sinchek K., Smiley N., York S. (2024). Assessing patient acceptance of an automated algorithm to identify ciswomen for HIV pre-exposure prophylaxis. J. Women’s Health.

[57] Hoover K.W., Zhu W., Gant Z.C., Delaney K.P., Wiener J., Carnes N., Thomas D., Weiser J., Huang Y.-L.A., Cheever L.W. (2022). HIV services and outcomes during the COVID-19 pandemic—United States, 2019–2021. Morb. Mortal. Wkly. Rep..

[58] Zhang C., Fiscella K., Przybylek S., Chang W., Liu Y. (2022). Telemedicine experience for PrEP care among PrEP-eligible women and their primary care providers during the first year of the COVID-19 pandemic in the United States. Trop. Med. Infect. Dis..

[59] Rice W., Logie C., Nápoles T., Walcott M., Batchelder A., Kempf M., Wingood G.M., Konkle-Parker D.J., Turan B., Wilson T.E. (2018). Perceptions of intersectional stigma among diverse women living with HIV in the United States. Soc. Sci. Med..

[60] Myers J.L. (2011). Why do young women get tested for sexually transmitted infections? Evidence from the National Longitudinal Study of Adolescent Health. J. Women’s Health.

[61] Noar S.M., Webb E., Van Stee S., Feist-Price S., Crosby R., Willoughby J.F., Troutman A. (2012). Sexual partnerships, risk behaviors, and condom use among low-income heterosexual African Americans: A qualitative study. Arch. Sex. Behav..

[62] Jennings L., Rompalo A.M., Wang J., Hughes J., Adimora A.A., Hodder S., Soto-Torres L.E., Frew P.M., Haley D.F. (2015). Prevalence and correlates of knowledge of male partner HIV testing and serostatus among African-American women living in high poverty, high HIV prevalence communities (HPTN 064). AIDS Behav..

[63] Lim S., Mantsios A., Braithwaite R., Pitts R. (2024). A secondary gendered analysis of interviews with Latina cisgender women indicated for HIV pre-exposure prophylaxis. AIDS Care.

[64] Cianelli R., De Santis J., De Oliveira G., Castro J., Iriarte E., Baeza M., Thomas S.O., Villegas N., Peragallo-Montano N. (2024). Feasibility and acceptability of SEPA+PrEP: An HIV prevention intervention to increase PrEP knowledge, initiation, and persistence among cisgender heterosexual Hispanic women. PLoS ONE.

[65] Calabrese S.K., Dovidio J.F., Tekeste M., Taggart T., Galvao R.W., Safon C.B., Willie T.C., Caldwell A., Kaplan C., Kershaw T.S. (2018). HIV pre-exposure prophylaxis stigma as a multidimensional barrier to uptake among women who attend planned parenthood. J. Acquir. Immune Defic. Syndr..

[66] Price D.M., Garretson M., Cai X., Miah F., Scanlin K., Blackstock O., Edelstein Z. (2024). Knowledge and attitudes about HIV pre-exposure prophylaxis among sexually active Black and Latina cisgender women: Findings from the 2017 and 2018 New York City sexual health survey. AIDS Patient Care STDs.

[67] Nydegger L.A., Kidane H., Benitez S., Yuan M., Claborn K.R. (2024). A qualitative exploration of PrEP interests, barriers, and interventions among Black and Latina cisgender women in the U.S. Arch. Sex. Behav..

[68] Chittamuru D., Frye V., Koblin B.A., Brawner B., Tieu H.V., Davis A., Teitelman A.M. (2020). PrEP stigma, HIV stigma, and intention to use PrEP among women in New York City and Philadelphia. Stigma Health.

[69] Matson P.A., Adler N.E., Millstein S.G., Tschann J.M., Ellen J.M. (2011). Developmental changes in condom use among urban adolescent females: Influence of partner context. J. Adolesc. Health.

[70] Lewis R., Mitchell K.R., Mercer C.H., Datta J., Jones K.G., Wellings K. (2020). Navigating new sexual partnerships in midlife: A socioecological perspective on factors shaping STI risk perceptions and practices. Sex. Transm. Infect..

[71] Zack J.L., Hull S.J., Coleman M., Ye P.P., Lotke P.S., Visconti A., Beverley J., Brant A., Moriarty P., Scott R.K. (2024). Age-related factors associated with intention to initiate pre-exposure prophylaxis among cisgender women in Washington D.C. Ther. Adv. Infect. Dis..

[72] Syme M.L., Cohn T.J., Barnack-Tavlaris J. (2017). A comparison of actual and perceived sexual risk among older adults. J. Sex Res..

[73] Smith T.K., Larson E.L. (2015). HIV sexual risk behavior in older Black women: A systematic review. Women’s Health Issues.

[74] Mancuso N., Weiss K., Siegler A.J., Sullivan P.S. (2026). Changes in geographic drive time access to pre-exposure prophylaxis (PrEP) in the United States, 2017 to 2025. Prev. Med. Rep..

[75] Harrington K.R.V., Chandra C., Alohan D.I., Cruz D., Young H.N., Siegler A.J., Crawford N.D. (2023). Examination of HIV preexposure prophylaxis need, availability, and potential pharmacy integration in the southeastern US. JAMA Netw. Open.

[76] Bond K.T., Gunn A., Williams P., Leonard N.R. (2022). Using an intersectional framework to understand the challenges of adopting pre-exposure prophylaxis (PrEP) among young adult Black women. Sex. Res. Soc. Policy.

[77] Abrams J.A., Odlum M., Tillett E., Haley D., Justman J., Hodder S., Vo L., O’Leary A., Frew P.M. (2020). Strategies for increasing impact, engagement, and accessibility in HIV prevention programs: Suggestions from women in urban high HIV burden counties in the eastern United States (HPTN 064). BMC Public Health.

[78] Jones J.L., Schwartz S., Kassanits J.E., Pyra M., Brewer R.A., Kao U., Blumenthal J., Rana A.I., Valeriano T., Benbow N.D. (2025). Rapid ART, rapid PrEP, and status neutral implementation in ryan white-funded clinics: Results from a multisite survey. J. Acquir. Immune Defic. Syndr..

[79] O’Connell H.R., Criniti S.M. (2021). The impact of HIV pre-exposure prophylaxis (PrEP) counseling on PrEP knowledge and attitudes among women seeking family planning care. J. Women’s Health.

[80] Stewart J., Ruiz-Mercado G., Sperring H., Pierre C.M., Assoumou S.A., Taylor J.L. (2024). Addressing unmet PrEP needs in women: Impact of a laboratory-driven protocol at an urban, essential hospital. Open Forum Infect. Dis..

[81] Goldberg A., Price D., Phi A., Ma M., Edelstein Z., Golub S. (2024). Increasing engagement in human immunodeficiency virus prevention among cisgender women in New York City with sexual health self-testing kits: A MaxDiff analysis. Sex. Trans. Dis..

[82] Meyer J.P., Lazarus E., Phillips K., Watts Z.T., Duroseau B., Carlson C., Price C.R., Kershaw T., Willie T.C. (2024). A PrEP decision aid for women survivors of intimate partner violence: Task-shifting implementation to domestic violence service settings. PLoS ONE.

[83] Giovenco D., Pettifor A., Powers K.A., Hightow-Weidman L., Pence B.W., Celum C., Delany-Moretlwe S., Hosek S., Donnell D., Anderson P.L. (2022). Intimate partner violence and oral HIV pre-exposure prophylaxis adherence among young African women. AIDS.

[84] Braksmajer A., Leblanc N.M., El-Bassel N., Urban M.A., McMahon J.M. (2019). Feasibility and acceptability of pre-exposure prophylaxis use among women in violent relationships. AIDS Care.

[85] Irie W.C., Mahone A., Johnson B., Marrazzo J., Mugavero M.J., Van Der Pol B., Elopre L. (2024). “Just the stigma associated with PrEP makes you feel like it’s HIV itself”: Exploring PrEP stigma, skepticism, and medical mistrust among Black cisgender women in urban and rural counties in the U.S. deep south. Arch. Sex. Behav..

[86] Scott R., Hull S.J., Kerrigan D., Wang Y., Pratt-Chapman M., Mathias-Prabhu T., Unwala N., Sadauskas M., Hose B.Z., Smith M. (2025). “The Women’s PrEP Project,” a clinic-based, socio-structural intervention to improve the provision of preexposure prophylaxis for cisgender women: Interrupted time series pilot. JMIR Form. Res..

[87] Chandler R., Guillaume D., Francis S., Xue E., Shah K., Parker A., Hernandez N. (2023). “I care about sex, I care about my health”: A mixed-methods pre-test of a HIV prevention mobile health app for Black women in the southern United States. PLoS ONE.

[88] Hill M., Mangum L., Coker S., Sutton T., Maria D.S. (2024). Dissemination and implementation approach to increasing access to local pre-exposure prophylaxis (PrEP) resources with Black cisgender women: Intervention study with vlogs shared on social media. JMIR Public Health Surveill..

[89] Allen D.C., Rabionet S.E., Dale S.K., Popovici I. (2025). What is the effect of medicaid expansions on preexposure prophylaxis for HIV prevention use among women?. AIDS Behav..

[90] Chandra C., Hamilton C., Young H.N., Holland D.P., Crawford N.D. (2026). Association between state policies enabling pharmacist-led human immunodeficiency virus pre-exposure prophylaxis and need for human immunodeficiency virus prevention: An ecological analysis. J. Am. Pharm. Assoc..

[91] Shrestha R.K., Hecht J., Chesson H.W. (2024). Analyzing the costs and impact of the takemehome program, a public-private partnership to deliver HIV self-test kits in the United States. J. Acquir. Immune Defic. Syndr..

[92] Knight D., Saleem H., Baral S., German D., Willie T. (2025). HIV pre-exposure prophylaxis (PrEP) modalities and service delivery preferences among Black cisgender emerging and older adult women in Baltimore, Maryland. BMC Public Health.

[93] Sharma A., Revees J., Heron K., Shangani S. (2025). Rural and urban differences in HIV pre-exposure prophylaxis (PrEP) awareness and acceptability among Black cisgender women living in the U.S. South. AIDS Care.

[94] Willie T.C., Knight D., Baral S.D., Chan P.A., Kershaw T., Mayer K.H., Stockman J.K., Adimora A.A., Monger M., Mena L.A. (2022). Where’s the “everyday Black woman”? An intersectional qualitative analysis of Black women’s decision-making regarding HIV pre-exposure prophylaxis (PrEP) in Mississippi. BMC Public Health.

